# Impact of the COVID-19 pandemic on TB infection testing

**DOI:** 10.5588/ijtld.21.0628

**Published:** 2022-02-01

**Authors:** V. Schiza, M. Kruse, Y. Xiao, S. Kar, K. Lovejoy, P. Wrighton-Smith, A. Tattersall

**Affiliations:** 1 Oxford Immunotec, Abingdon, UK; 2 Oxford Immunotec, Marlborough, MA, USA

Dear Editor,

We report here on the detrimental impact that the COVID-19 pandemic has had on testing for TB infection, with a sharp decrease in tests performed at the primary UK referral laboratory following the first pandemic-related lockdown. The Oxford Diagnostic Laboratories (ODL) performs a variety of tests, including using the T-SPOT.*TB* test (Oxford Immunotec, Abingdon, UK), a standardised ELISpot platform, to detect interferon-gamma (IFN-γ) release from immune T-cells after exposure to *Mycobacterium tuberculosis*.^1^ This is one of two IFN-γ release assays (IGRAs) in widespread use for detecting TB infection. Following the lockdown in late March 2020, we experienced a noticeable reduction in the number of T-SPOT.*TB* tests received for analysis (Figure A). As a decline in TB infection testing is an ominous sign for achieving the End TB goals,^2^–^5^ we investigated the difference between the expected vs. observed number of TB tests performed over the period April 2020–August 2021, which included three lockdowns in the United Kingdom. Data for this retrospective study were obtained in accordance with UK privacy regulations and the ODL Confidentiality Policy.

The number of tests performed from January 2017 to March 2020 at ODL followed a generally increasing trend (Figure A). However, there was a 73% decrease in the number of tests performed from March to April 2020. Although testing increased in subsequent months, it had not returned to prelockdown levels by August 2021. To estimate the extent of this decline from April 2020 to August 2021, we first calculated a linear best-fit line for data from January 2017 to March 2020 and projected this best-fit line forward until August 2021 (Figure A, dotted line). We then subtracted the actual number of tests performed from the expected (projected) number for each month from April 2020 to August 2021. Our analysis indicated that 41% of expected TB infection tests were not performed during the intermittent pandemic lockdowns from April 2020 to August 2021. We next explored the source of the test samples to see which sources decreased the most during the pandemic. The samples for analysis come from three sources: 1) routine testing of suspected TB patients and occupational health screenings, 2) contact tracing of known TB disease cases and group screening events, and 3) NHS England’s national tender testing of 16–35-year-olds recently arrived from high TB incidence countries (defined as countries with a TB rate of ≥150/100,000). A sharp decrease in tests from all three sources was observed during the pandemic, especially in April, May and June 2020 following the first lockdown (Figure B). In particular, the number of contact tracing and national tender tests, which accounted for respectively 7% and 11% of pre-pandemic testing, practically disappeared during these 3 months. Routine testing, which represented 82% of pre-lockdown testing, essentially halved in the 3 months after the first lockdown. However, due to the almost complete elimination of the other two categories, routine testing represented 97% of the TB testing being done during the 3 months after the first lockdown. Routine testing rebounded to almost pre-lockdown levels by August 2021, whereas the other two categories remained below pre-lockdown levels (Figure B).

The initial drop in the national tender testing can be attributed to NHS England pausing TB infection screening of recent arrivals in March 2020,^6^ in parallel with the UK Government’s COVID-19-related travel restrictions. These restrictions likely reduced the arrival of people with potential TB infection; the decrease in the national tender testing may therefore not reflect an increased TB risk in the United Kingdom in the short term. However, the decrease in routine testing and contact tracing may have increased the risk for future TB cases. Although the lockdown limited large gatherings, and so reduced the risk of TB transmission outside of households, lockdowns could increase TB transmission within households. Furthermore, a fear of contracting COVID-19 in health facilities may have discouraged people from seeking medical help.^7^–^10^ Also, the diversion of healthcare resources, facilities and infectious disease personnel to the fight against COVID-19^2^,^7^,^9^ may have led to fewer TB tests being ordered. In addition, the symptoms of pulmonary TB and COVID-19 can overlap, raising the possibility of undiagnosed TB infections without proper vigilance.^11^

These results show that the most severe disruption in TB infection testing occurred from April to June 2020, when the first lockdown was introduced. This mirrors TB notifications in England, which decreased by 24.6% from April–June 2020 compared to the same months of 2019.^12^ To address this threat to public health, the 2020 TB report by Public Health England noted the need to improve “TB infection (LTBI) testing and treatment to prevent reactivation of TB and transmission, a service that has been greatly affected by COVID-19”.^4^ This assessment for the United Kingdom mirrors the worldwide assessment. The significant decrease in TB infection testing during the COVID-19 pandemic reported by many countries^5^,^9^,^13^–^15^ suggests that the pandemic could increase the global TB burden. Indeed, the Stop TB Partnership estimated that in addition to the prepandemic number of 1.5 million TB deaths each year,^2^ the net effect of the COVID-19 pandemic could be an additional 6.3 million TB infections and 1.4 million TB deaths between 2020 and 2025.^3^

**Figure:**
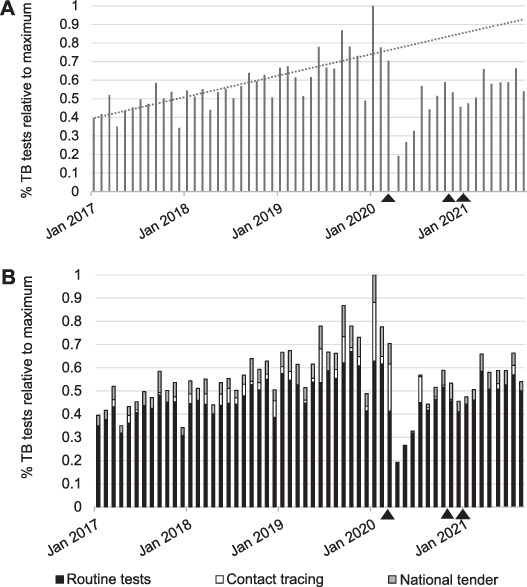
The percentage of T-SPOT.TB tests performed at the UK Oxford Diagnostic Laboratories from January 2017 to August 2021. A) The percentage of tests per month relative to the maximum number of tests performed in the time period. The dotted line is the linear best-fit to the time period from January 2017 to March 2020; the best-fit line is projected to cover the period April 2020–August 2021. B) The percentage of tests by test category: routine testing (lower gray bars), contact tracing (middle black bars) and national tender (upper white bars). Black triangles indicate the three UK lockdowns, which began on 26 March 2020, 5 November 2020 and 6 January 2021.

A limitation of this study is that it only describes the changes to testing with T-SPOT.*TB*. Two IGRAs are available for TB infection testing in the United Kingdom, the T-SPOT.*TB* test and the QuantiFERON^™^-Plus test (Qiagen, Hilden, Germany). As we did not have access to equivalent test volume data for the QuantiFERON-Plus test, we cannot generalise our results to all TB infection tests conducted in the United Kingdom. However, our findings are consistent with other reports.^5^,^9^,^13^–^15^

Efficient diagnosis and treatment for TB is necessary to decrease community transmission and the burden of TB.^2^–^4^ Thus, ending the global TB epidemic will require not just a return to normal TB infection screening but expanded efforts to mitigate these TB cases undiagnosed during the pandemic.^3^ In addition, consideration could be given to modifying TB guidelines to address the need for continued TB infection screening during any future lockdowns.
